# Suggestions from a Co-laborer

**Published:** 1876-12

**Authors:** 


*Suggestions from a Co-laborer.—Messrs. Editors:* I have received your circular announcing your determination to adopt the cash system in the futute. As a friend to your enterprise, and as one who has had experience in a similar undertaking, allow me to make two or three suggestions for your consideration.

Your adoption of the cash system is wise and proper. It ought to have been done from the start. Experience has settled the point, beyond all controversy, that *no journal can live, on any other plan.* I heartily accept the change, and do hope that every subscriber will feel as I do about it, and will renew his subscription. Your journal deserves to be sustained. I think I speak the sentiments of a very large majority of those who read it, when I say it is the most practical journal in this country, and exceedingly useful to the busy practitioner. Your *Extracts* and *Gleanings*, for brevity, variety and useful suggestions and formulas surpasses any journal I have ever seen. And your original department, though occasionally burdened with too long an article, has in the main been well sustained. And this brings me to another suggestion, which is this—*reduce the size and price of your journal*, and it will have still greater success, and may, in my humble judgment, be made equally as acceptable to most of your readers as at present—the original department being condensed, and the whole journal being made to conform to the excellent motto under which you sail—which if I rightly interpret it, means *brevity*. In your "Selections" you may cut off much by making abstracts, and in this way may cut down expenses, increase rather than diminish the interest of your journal, and by a lower subscription will obtain large additions to your list of subscribers.

